# An interconnect-free micro-electromechanical 7-bit arithmetic device for multi-operand programmable computing

**DOI:** 10.1038/s41378-023-00508-0

**Published:** 2023-04-03

**Authors:** Xuecui Zou, Usman Yaqoob, Sally Ahmed, Yue Wang, Khaled Nabil Salama, Hossein Fariborzi

**Affiliations:** CEMSE Division, King Abdullah University of Science and Technology, Thuwal, 23955 Saudi Arabia

**Keywords:** Electrical and electronic engineering, Engineering

## Abstract

Computational power density and interconnection between transistors have grown to be the dominant challenges for the continued scaling of complementary metal–oxide–semiconductor (CMOS) technology due to limited integration density and computing power. Herein, we designed a novel, hardware-efficient, interconnect-free microelectromechanical 7:3 compressor using three microbeam resonators. Each resonator is configured with seven equal-weighted inputs and multiple driven frequencies, thus defining the transformation rules for transmitting resonance frequency to binary outputs, performing summation operations, and displaying outputs in compact binary format. The device achieves low power consumption and excellent switching reliability even after 3 × 10^3^ repeated cycles. These performance improvements, including enhanced computational power capacity and hardware efficiency, are paramount for moderately downscaling devices. Finally, our proposed paradigm shift for circuit design provides an attractive alternative to traditional electronic digital computing and paves the way for multioperand programmable computing based on electromechanical systems.

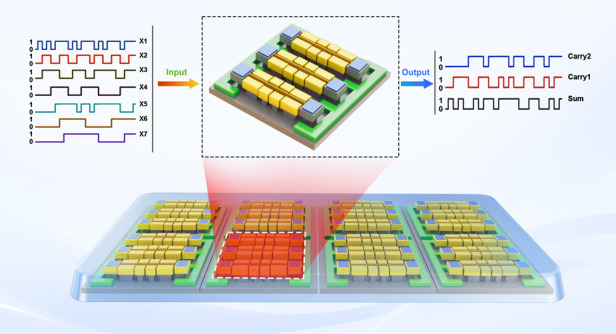

## Introduction

Mechanical mechanisms for processing information can be traced back to the analytical machines of Charles Babbage in 1822^1^. More recently, subsequent developments in complementary metal–oxide–semiconductor (CMOS) technology have outperformed these mechanical forms in computation due to better capabilities for speed of operation and miniaturization^2^. However, miniaturization is rapidly approaching its physical limits and the energy efficiency of highly miniaturized devices is degrading^3^. With manufacturing capabilities reaching the molecular limit^4^ through advances in materials and structural engineering^5^, interest in electromechanical computing has been revitalized as a method for replacing conventional electronics^6–8^.

Unlike MEMS/NEMS relays operated in the contact switching mode^9^, new dynamic MEMS/NEMS computing methodologies (operating based on vibration amplitude or phase) are being vigorously developed and are considered to be free from contact reliability issues, contact resistance, surface stiction, and mechanical delay limitations^10–12^. Moreover, ultralow power consumption^13^ and the potential for hardware reprogrammability^14^ and computation reversibility^15–17^ have made MEMS/NEMS resonators appealing alternatives to existing information processing technologies. Although there have been successful demonstrations of memory components^18–20^ and 2- or 3-bit logic gates using MEMS resonators^21–23^, the realization of complex multi-input logic circuits with multioperand for high computation capacity has remained a challenge. In addition, the concepts of combining hardware reprogrammability and encoding multiple signals with different frequencies to allow enhanced functionality and aggressive complexity reduction have not yet been thoroughly explored.

Herein, we thus present a microbeam resonator-based 7-bit accumulator and binary coder (7-3 compressor), e.g., a compulsory arithmetic component for computing systems and digital microprocessor chips, which can execute dedicated algorithms such as partial product reduction and information compression. Then, an m-n/m:n compressor, where m and n denote inputs and outputs bits that accumulate the logic inputs, suppresses the input bit into fewer output bits without sacrificing precision tolerance. The proposed 7-3 compressor consists of three parallel-placed resonators encoded with multiple signals of specific frequencies for efficient and accurate compression. Multiple input frequency channels of binary information are synthetically applied to activate specific mechanical oscillations and assure the construction of high computational power capacity and flexibility of arithmetic functionality. Moreover, the performance metrics such as signal-to-noise ratio, switching reliability, and bias instability are evaluated following device optimization. Finally, performance parameters such as energy consumption, device count, and hardware efficiency are compared with existing technologies.

## Results

### Finite element analysis for device optimization

**Fig. 1:**
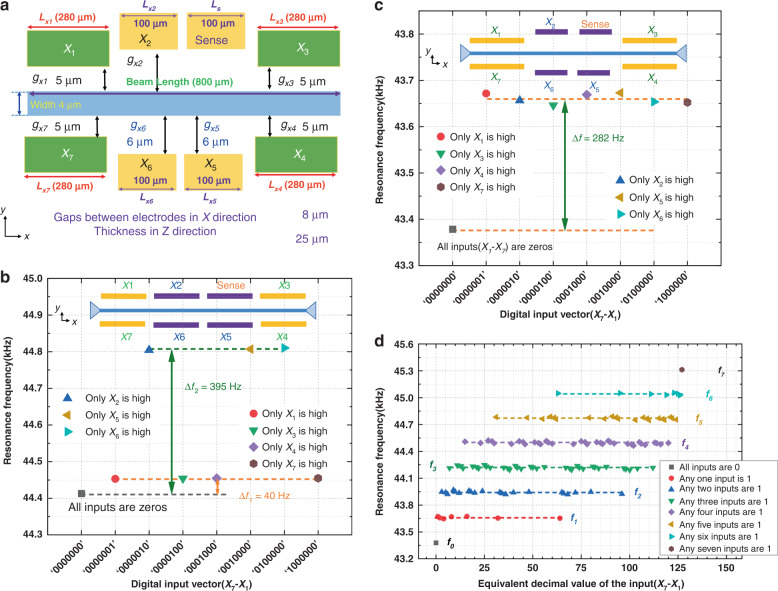
Finite element analysis of the compressor. **a** Schematic of the device with optimized structural parameters. **b** Stiffness modulations of the prototype. **c** Stiffness modulations of the optimized compressor. **d** Resonance frequencies are grouped by the number of input ‘1’s; the equivalent decimal value (0 to 127) of the digital input (000-0000 to 111-1111) is used for the x-axis label

To design an interconnect-free compressor, optimizing the number of electrodes, dimensions, and structural configuration is of great importance. Figure 1a shows the top view of the microbeam structure with eight electrodes and their optimized dimensions. Finite element analyses are carried out to successfully optimize the dimensions and configuration for all eight electrodes prior to building the 7:3 compressor.

The COMSOL Multiphysics simulation is performed using the Electro-Mechanics Physics and Structural Mechanics modules to investigate DC modulation effects during the optimization of the device structures. We set the input voltages to 20 V for a logic ‘1’ and 0 V for ‘0’. For optimization, we conduct a simulation where all eight partial electrodes have the same air gap distance (6 µm) from the microbeam; the inset of Fig. 1b shows a schematic of this electrode configuration. The simulation results are carried out by sequentially exciting each electrode (*X*_1_–*X*_7_) at a time to calculate the shift in resonance frequency (Fig. 1b). For this configuration, the change in resonance frequency is not consistent when applying high inputs. It is observed that electrodes *X*_2_, *X*_5_, and *X*_6_ demonstrate greater control over the resonance frequency (shift ~395 Hz), while *X*_1_, *X*_3_, *X*_4_, and *X*_7_ possess less control (shift ~40 Hz), suggesting that electrode optimization is required (Fig. 1b). It is critical to design these electrodes in a configuration where the same level of frequency shift can be achieved with any case of single high input. Figure 1c illustrates the simulation results for the optimized configuration. At any high input, the level of resonance frequency shift remains almost constant, indicating its capability to accurately perform the logic operation. Therefore, by switching the digital input in the electrodes *X*_7_–*X*_1_ from 000-0000 (the equivalent decimal is 0), …, 101-1011 (the equivalent decimal is 91), …, to 111-1111 (the equivalent decimal is 127), where 20 V establishes logic ‘1’ and 0 V establishes ‘0’, the frequency response of the optimized resonator is obtained in Fig. 1d. The parametric sweep on the seven digital inputs to obtain the one-to-one relationship between the summation of input ‘1’s and the corresponding resonance frequency is shown in Fig. 1d. Thus, our proposed design is capable of summing the number of input ‘1’s appearing in the seven digital input bits from 000-0000, …, 101-1011, …, to 111-1111 and expressing their summation as the corresponding frequency values (*f*_0_–*f*_7_). The working mechanism and experimental configuration for the proposed 7:3 compressor are detailed in the following section.

**Fig. 2:**
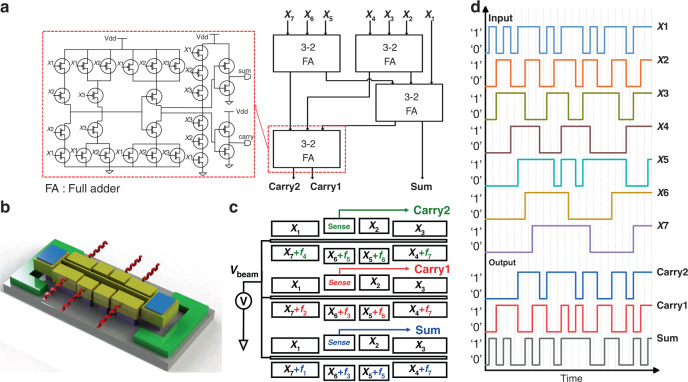
Schematic diagram of the 7-3 comsorpres. **a** Traditional CMOS-based compressor structure. **b** Structural diagram of the resonator. **c** System schematic of the 7-3 compressor. **d** Time domain functional responses of the output Carry2 to Sum regarding the *X*_7_−*X*_1_ inputs

A conventional compression structure is the 7-3 compressor, which comprises four cascaded full-adder (FA) modules, as shown in Fig. 2a. As the name suggests, its working functionality is to compress the seven input operands (*X*_1_ to *X*_7_) to only three outputs in binary format (Carry2, Carry1, and Sum). For example, a 7-3 compressor that takes input bits of ‘0000101’, including two high inputs, is expected to generate output signals in a binary format with 3 bits, ‘010’. The output of a 7:3 compressor is derived using Eq. 1.

1 $$X_1 + X_2 + X_3 + X_4 + X_5 + X_6 + X_7 = 2^2 \cdot {\rm{Carry}}2 + 2^1 \cdot {\rm{Carry}}1 + 2^0 \cdot {\rm{Sum}}$$

The seven inputs (*X*_1_ to *X*_7_) of a 7-3 compressor are equally weighted, and their summation is represented by the Carry2, Carry1, and Sum bits.

### Resonator-based compressor and experimental principle

The proposed 7:3 compressor consists of an array of three parallel identical resonator units driven with specific frequencies to generate the three-bit binary output (Carry1, Carry2, and Sum). The structural schematic of a single resonator unit with eight partial electrodes is displayed in Fig. 2b. All three units consist of eight partial electrodes for I/O interfaces, a clamped-clamped bridge structure, and one external blanked metal pad, which is posited outside the resonators to ground the whole chip.

Resonators are driven by the harmonic electrostatic force generated by the polarized voltage between the electrodes and the beam through parallel overlapped capacitors. Seven input electrodes (*X*_1_–*X*_7_) are used to load electrostatic modulations on the resonance frequency of the microbeam, and the scale is optimized through electrode configurations to obtain seven equally weighted inputs. The logic input switches the voltage polarization between the input electrode and the resonator. Thus, the induced electrostatic force loaded on the resonator affects its static deflection and stiffness perturbation. The essential principle of the resonator (to perform compression operations) relies on these switch-induced stiffness perturbations.

Three parallelly mounted resonators are required to obtain the 3-bit binary outputs using multiple frequency-driven techniques. Table 1 summarizes the binary output mechanism and digital operand-frequency relationship. The single resonator unit driven by specific combined frequencies of the designated input combinations is capable of achieving flexible complex operations. For instance, to perform a sum operation, we operate a resonator with specially assigned frequencies *f*_1_, *f*_*3*_, *f*_5_, and *f*_7_ that correspond to the *f*_*n*_ (sum equals 1) combinations, as shown in Fig. 2c. These specially assigned frequencies are exclusively valid when assuming that each input frequency (*f*_0_–*f*_7_) is linked with different combinations of input operands to perform the summation function, consistent with the simulation results shown in Fig. 1d for the optimized structure. The oscillatory displacement amplitudes of high and low states of the resonator are reflected as high and low values of the transmitted signal, indicating the digital high and low output of the device. Therefore, to execute a 7:3 compressor operation, each resonator (Carry2, Carry1, and Sum) is operated using multiple assigned frequencies, as shown in Fig. 2c.

**Table 1:** The truth table of the proposed 7:3 Compressor

$\mathop {\sum }\limits_{i = 1}^7 X_i$	Resonance frequency	Outputs			Input combination/All combinations
		Carry2	Carry1	Sum	
0	*f*_0_	0	0	0	1/128
1	*f*_1_	0	0	1	7/128
2	*f*_2_	0	1	0	21/128
3	*f*_3_	0	1	1	35/128
4	*f*_4_	1	0	0	35/128
5	*f*_5_	1	0	1	21/128
6	*f*_6_	1	1	0	7/128
7	*f*_7_	1	1	1	1/128

The multifrequency-driven signals cause the beam to vibrate with a large amplitude when any one of the driven frequencies of the applied signal matches its resonance frequency. As shown in Fig. 2d, the output amplitudes of the three resonators switch under seven inputs, demonstrating that the resonators driven by four driving frequencies can convert the equal-weighted inputs into binary outputs. Using independent ports to implement the AC signal of each specific frequency eliminates the frequency interference factor and the need for any frequency mixing component.

**Fig. 3:**
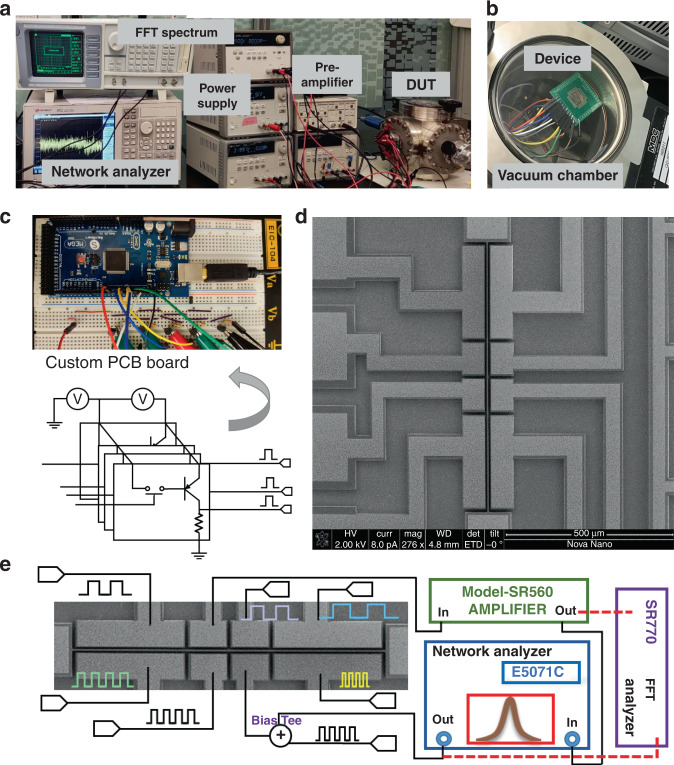
Experimental scheme of the compressor. **a** Photo of the experimental setup. **b** Photo of the device under test. **c** Customized PCB board for synchronous input control. **d** SEM photograph of the resonator unit. **e** Schematic diagram of the open-loop setup

An experimental scheme of the two-port electrical transmission measurement configuration for electrostatic actuation and capacitive sensing of the resonator is displayed in Fig. 3a. The device under test (DUT) is placed in a vacuum chamber at a pressure of 300 mTorr to limit the nonlinear vibrations. The driving ports are provided with an AC + DC signal from the output of a passive bias tee diplexer (BT-1510-B), and the beam electrode is biased with a DC voltage source. The output current induced at the sensing electrode is converted and amplified using the low-noise amplifier, which is then connected to the input port of the network analyzer. Seven inputs of the compressor circuits are controlled and synchronized using the customized PCB. The customized PCB consists of bipolar junction transistors and resistors connected in parallel across the output pins of an Arduino Mega 2650-board, as shown in Fig. 3c. The digital input (switch ON/OFF condition) to input channels *X*_1_–*X*_7_ is modulated by the voltage High/Low of the respective electrical pin, which respectively corresponds to the binary values (1/0).

The functional characterization setup is depicted in Fig. 3e. The compressor unit is operated directly in analogy to transistor-based compressors: when the bias voltage is applied to the beam, the resonant channel begins working, and the polarized voltage states of the digital inputs change the amplitude at the output terminal. The logic output is collected from the sensing port, where a relatively high (low) *S*_21_ transmission signal corresponds to the logic output 1 (0). A Stanford FFT spectrum analyzer (SR770) provides a triggering signal for characterizing the noise at the system level in the open-loop measurement configuration.

### Device characterizations

#### Operational frequencies and standard deviations

**Fig. 4:**
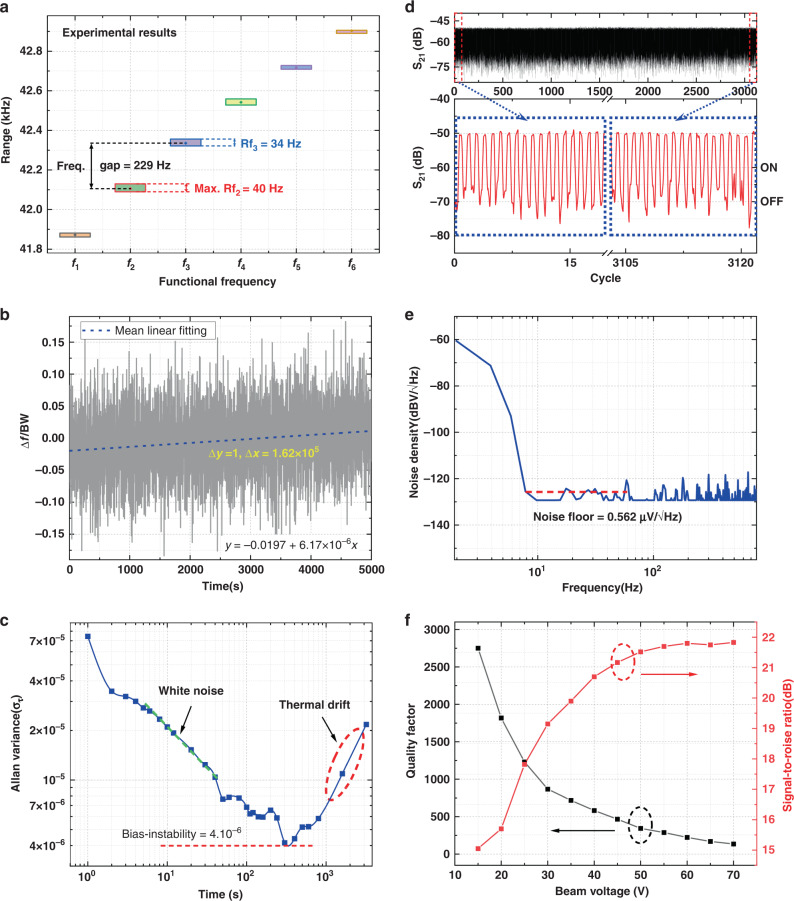
Experimental analysis of the device. **a** Frequency range of each group (*f*_1_−*f*_7_). **b** Long-term frequency drift testing results in transient. **c** Standard Allan variance analysis. **d** Switching on/off operation with thousands of cycles. **e** Output noise density spectrum. **f** Switching speed and signal-to-noise ratio versus beam bias voltage

It is of extreme significance to experimentally test a range of frequency differences for each group to identify the compressor functionality, including different input operand combinations. The frequency variations for each group are evaluated using Eq. 2:

2 $$Rf_n = f_{{\rm{max}},n} - f_{{\rm{min}},n}$$

where *f*_max,*n*_ (maximum frequency) and *f*_min,*n*_ (minimum frequency) belong to the same frequency category *f*_*n*_. Figure 4a shows that the maximum frequency variation range occurs at *f*_*2*_, *Rf*_2_ = 40 Hz, which is relatively higher than *Rf*_*3*_ (34 Hz). If the frequency variation range for each group reaches *Rf*_*n*_ > 114 Hz (half of the frequency gap), the compressor may fail. Therefore, the maximum frequency variation range is only 40 Hz for group *f*_2_, which meets the requirement to perform a successful compression operation.

Figure 4b records the transient frequency drift at switch-on states carried out for over 5000 s to reveal the frequency stability of the device. The output is measured at a sampling rate of 10 Hz. It is suspected that an erroneous output might be generated when the frequency drift is greater than the half-power bandwidth (BW) of the resonator. Accordingly, the ratio of the measured frequency drift and BW is linearly fitted and described by the following equation:

3 $$\frac{{\Delta f}}{{BW}} = - 0.0197 + 6.17 \times 10^{ - 6}t$$

According to the mean linear fitting results, the slope of the frequency drift over time is calculated to be ~6.17 × 10^−6^, suggesting that the device can perform correct operations consistently for at least *t* ~ 45 h.

The frequency instability is obtained by performing a standard Allan derivation on the acquired data through data processing in MATLAB. According to the effective output signals from the resonator and amplification factor of the circuit, a modified Allan variance of the dataset can be calculated to assess the resonance frequency. According to the standard 1-h Allan variance analysis, the frequency fluctuations can be classified into three main components: white noise, bias instability, and thermal drift. The device’s white noise and thermal drift can affect the compressor operation accuracy^24^. Bias instability is mainly contributed by flicker noise and refers to the random variation in the calculated deviation within a specified finite sampling time and average time interval. Figure 4c displays the bias instability of the device, which is ~4 ppm across an averaging timespan of 300 s.

The measured Allan derivation describes the frequency fluctuations in the resonator, which affects the bit error of the output signal. The bit error can be calculated via the following formula:

4 $$Er = \frac{{\sigma _\tau }}{{BW/f}} = 2.8 \times 10^{ - 3}$$

Considering the tolerance of a typical digital computing unit (0.1)^25^, our device provides a tolerable bit error rate and exhibits acceptable reliability for computing.

#### Long-term switching reliability

To study the switching reliability, the AC driving frequency is set at *f*_1_ = 41.87 kHz for continuous switching (on/off) with ~3000 repeated cycles. For this set condition, a high S_21_ transmission signal (logic output 1) is achieved for single high input (switch ON), while for other combinations, it remains 0. The time width for each state is 0.78 s. The switching operations for the device under thousands of repeated cycles are tested and depicted in Fig. 4d. No failures in the switching operations of the device are observed for all of the 3000+ cycles, showing the excellent and stable switching capability of our proposed device. The reliability of the system and the repeatability of the switching action between the two output states are guaranteed.

#### Noise power, quality factor, and SNR analysis

The working of a device is achieved using a single block of an oscillating unit. The device’s switching speed is limited by the characteristic time scale of energy exchange between the vibrated motion at the resonance frequency (ω_r_) and its surroundings. Under the open-loop scheme, the mechanical transition time is limited by the quality factor (~700), which can be evaluated using *Q*/*ω*_*r*_ ~ 0.016 s^13^. It is important to mention another time constant: the time required to charge/discharge the parallel plate capacitance between the beam and the partial electrodes while switching digital inputs. In addition, by leveraging all digital inputs’ synchronous operation, the maximum delay is decreased from 6.1 ps to 1.2 ps, which negligibly contributes to the total switching speed. A fast Fourier transform analyzer (SR770) is used to record the output noise of resonators based on an open-loop control scheme. With the cutoff frequency of the proposed compressor being 60 Hz, the noise power spectral density (PSD) of the compressor in Fig. 4e shows that the noise floor of the resonator is 0.56 µV/√Hz.

The air gaps between the beam and electrodes are also affected by static deflection, which can be utilized to modulate the quality factor of the driven micro-resonator. Thus, the beam bias can also modulate the switching speed of the compressor. To improve the switching speed, the quality factor can be directly reduced by decreasing the vacuum level of the testing environment. However, the increased vacuum pressure adversely influences the SNR of the output. As displayed in Fig. 4f, the variation in the quality factor is approximately logarithmically decreased with beam bias voltages ranging from 15 V to 70 V. Notably, the maximum operational speed of 240 Hz is achievable in this submillimeter-scaled resonator. In addition, the speed can be increased significantly by downscaling the resonator structure to achieve higher resonant frequencies (megahertz or gigahertz), as shown in Table 2.

**Table 2:** Delay, area, and power consumption of the proposed micro-resonator design with different dimensions, including Design 1 (experimental results) and Designs 2–4 (simulated results)

Reso.	Dimensions			Performance			
	Length	Width	Depth	Frequency	Delay	Energy/op	Area
Design 1	800 μm	4 μm	25 μm	41.3 kHz	16 ms	20.73 pJ	12,800 μm^2^
Design 2	180 μm	1.2 μm	5 μm	278.4 kHz	0.36 ms	0.74 pJ	864 μm^2^
Design 3	12 µm	0.4 μm	0.8 μm	21.5 MHz	4.65 μs	69.6 fJ	19.2 μm^2^
Design 4	1.5 µm	120 nm	120 nm	423 MHz	240 ns	1.14 fJ	0.72 μm^2^

The signal-to-noise ratio is another essential metric for logic devices that reports the output quality to evaluate how this output signal is disturbed by the noise. The measured results provide us confidence that the proposed compressor produces SNRs of more than 15 dB, which fully satisfy the requirements for image processing applications^26^. In addition, with the increased operating beam bias voltage, the SNR of the compressor can be efficiently improved from 15 dB to 22 dB, which achieves higher quality outputs at the cost of energy efficiency.

### Functional verification

**Fig. 5:**
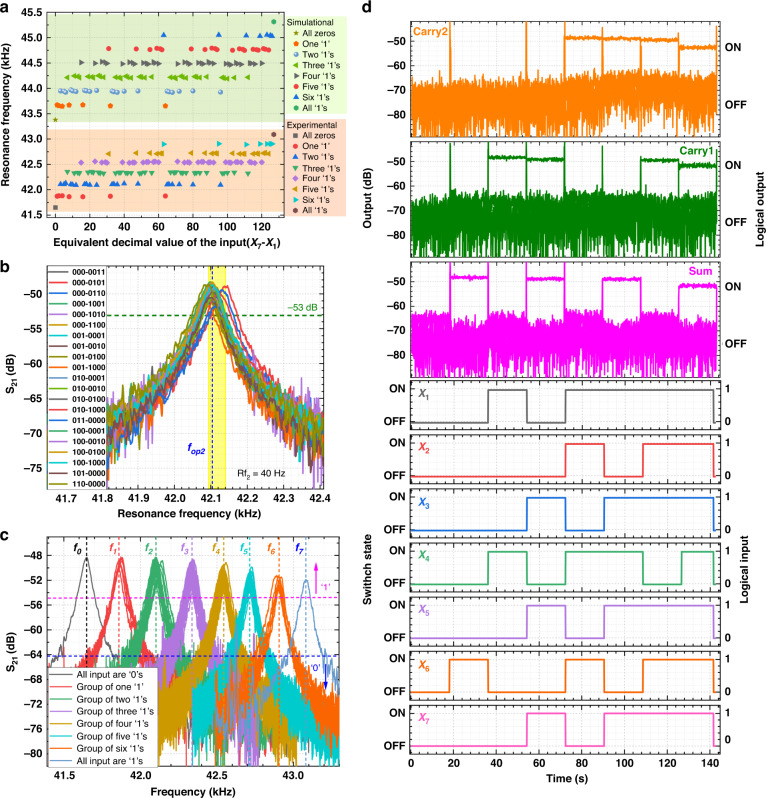
Experimental verification of the compression function. **a** The relation between the resonant frequencies and the summation of digital inputs. **b** Frequency responses at *f*_2_. **c** frequency responses at *f*_0_‒*f*_7_. **d** Transient outputs of the resonator

The compressor is activated to detect resonant frequencies under all 128 combinations of input vectors (*X*_1_–*X*_7_ from 0000000 to 1111111). The measured frequency values are recorded and compared with the simulation results, as shown in Fig. 5a. With the stiffness modulation effects, the 128 resonant frequencies can be grouped into eight categories. The frequencies of each category appear to be at almost the same level because each input has the same loading on the resonance frequency. The slight deviation between the simulation and experiment is attributed to residual stresses and fabrication tolerance. The established function of the resonant frequency with the number of inputs ‘1’s exhibits the same trend in the simulation and experimental results.

#### Frequency response

Forward- and backward-frequency sweeps were performed and ensure that the compressor is always working in the mono-stable regime (where only one output solution is accessible to avoid failure of an operation). The frequency responses measured under an open-loop scheme without asymmetry in the magnitude also indicate no bistability of the vibration motion. Furthermore, when the bias voltage was applied to the beam, the resonant channel from the driving port to the sensing port started working. The amplitude at the sensing terminal depended on the mutual interaction between driving frequencies and the resonance tuned by the digital input vectors. If the driving frequencies were far from the resonant frequency, the amplitude on the sensing electrode would be low, resulting in a relatively low *S*_21_ transmission signal (<−64 dB) and logic ‘0’ output (Fig. 5b). Alternatively, when any one of the driving frequencies matches the resonant frequency, a relatively higher *S*_21_ transmission signal (>−55 dB) will be received at the sensing electrode, thus the displaying logic ‘1’ output.

As shown in Fig. 5c, traversing all combinations containing two high inputs (‘1’s), the frequency responses are clustered together with a maximum frequency difference of Rf_2_ ~ 40 Hz. Thus, for the device operated at *f*_op2_ = 42.104 kHz (marked by the blue dashed lines), the biased compressor would output a relatively higher *S*_21_ transmission signal (>‒53 dB) and generate the logic output ‘1’ with any condition of summation for two high inputs. Note that the amplitudes from *f*_0_ (7 input ‘0’s) to *f*_7_ (7 input ‘1’s) show a slight magnitude reduction because the more input operands at high states, the lower the force amplitudes induced on the beam are^27^.

#### Time responses

There are 128 input combinations for seven-input digital devices, resulting in 8 different levels of resonant frequencies. For a resonator driven with *f*_op2_, the amplitude in the sensing terminal depends on whether the digital input vectors contain two high inputs. The resonator, as driven by multiple frequencies, opens the channel for digital input vectors containing different combinations of high input vectors. Table 1 shows the frequency combination for obtaining each bit of the binary output compressor. For example, the resonator with Carry2 output, driven by *f*_4_*, f*_5_*, f*_6_, and *f*_7_, opens the resonant channel only for the input vectors that have four, five, six, and seven high inputs. The high S_21_ amplitude only occurs at a specific frequency because only one driving frequency can open the channel at any given time.

Therefore, we obtain other digital output bits by correctly choosing and combining the AC driving frequencies. The operating frequencies *f*_1_*–f*_7_ are combined and assigned to the compressor resonators to obtain different logic operations. Finally, compression operations are successfully achieved by selecting the appropriate AC driving frequencies using our optimized system. Figure 5d shows the dynamic response in the time domain of the conceived resonator system representing the summation of digital inputs in a binary format.

### Performance comparison matrix

Energy consumption is one of the critical parameters regarding the performance of our proposed compressor. The energy consumed by the resonator comprises two parts: switching energy (*E*_switch_), which is consumed when the digital inputs change their voltage value(s), and the actuation energy, which is consumed by the AC signal applied at the driving electrode (*E*_AC_). The switching energy depends on the input pattern applied to the circuit, which will either cause the resonators to switch (charging/discharging capacitances) or to maintain the previous state (no power consumption) at each clock period. All possible input combinations for the compressor were considered (2^7^ = 128) to obtain the probability of energy consumption at each input combination. With the assumption that all inputs have the same possibility for ‘1’ and ‘0’, the switching energy can be given using the following equation:

5 $$E_{{\rm{switch}}} = \mathop {\sum }\limits_{m = 0}^7 \frac{1}{2}p_mmC_{{\rm{ave}}}\left| {\Delta V_{{\rm{ON}}}^2 - \Delta V_{{\rm{OFF}}}^2} \right|$$

where *p*_*m*_ is the probability of the input combinations that the summation of inputs equals *m*, and *C*_ave_ is the average capacitance between the beam and the electrodes. The capacitances are not the same for inputs *X*_1,3,4,7_ and *X*_2,5,6_ due to the different air gap and electrode length values for the corner and center electrodes, respectively. *∆V*_ON_ and *∆V*_OFF_ are the polarized voltage difference between the beam and the electrode: the digital inputs are either 0 V for digital ‘0’ or 9 V for digital ‘1’, resulting in a *∆V*_ON_ of 35 V and *∆V*_OFF_ of 24 V. The switching energy is calculated by taking the average of all possible combinations (2^7^ = 128), which is estimated to be approximately 4.9 pico-joules per conversion step for all three resonators.

The other component of the energy consumption is the *E*_AC_, dissipated during the beam vibration motion, which can be conservatively estimated based on the method described in^28^. *E*_AC_ depends on the amplitude of the AC drive signal and the effective impedance of the resonator. This resonator impedance includes the motional resistance and the parasitic impedance between the driving and sensing electrodes, which is measured to be 397.2 kΩ by terminating the resonator output directly to the network analyzer:

6 $$E_{{\rm{AC}}} = \frac{{V_{{\rm{ac}}}^2}}{R}\frac{Q}{{\omega _r}}$$

where *V*_ac_ = 7.07 mV is the AC driving voltage amplitude. Thus, one resonator consumes 2.01 pJ per driving logic operation. Each compressor system requires three resonators to generate a 3-bit logic output; therefore, the total energy consumption is 20.73 pJ per conversion step. Notably, the AC driving energy can be further minimized to compensate for the power dissipation of the vibrating motion when the resonator is at a stable logic status. The thermal fluctuations of the system for energy change between the resonator and its surroundings to determine the minimum power level dissipated by the vibration mode^29,30^, which can be estimated by:

7 $$P_{{\rm{stable}}} = k_BT\ln 2 \cdot \frac{Q}{{\omega _r}} = 6.62 \times 10^{ - 23}W$$

At room temperature, the minimum leakage power is approximately 6.62 × 10^−23^ watts for static power consumption.

By optimizing the design, from the material and structure to the device dimensions, a resonance frequency of >100 MHz is attained^31^. In Table 2, the reduced device dimensions of simulated Designs 2–4 result in higher resonance frequencies, leading to a lower delay and faster operating speed. A compressor with a minimum critical parameter of 120 nm (Design 4) is expected to consume 1.14 fJ/conversion step with an operation speed of 4.2 MHz, assuming a Q of 100. Table 2 reveals the energy per operation, delay, and area shrinkage with downscaling structural dimensions. Compared to the tested device (Design 1), the nanoscale structural scale reduces the energy per operation by orders of magnitude, and a delay of nanoseconds is attainable. In addition, the reduction in gap distance between the electrodes and the beam enables the use of lower DC voltages for digital inputs and beam bias.

It is worth mentioning that the 7-bit data compressor can be implemented using three resonator units for the proposed technique, which outperforms the existing resonator-based compressor’s computational capability. The figure of merit to evaluate the computational capability per unit can be defined using the following equation:

8 $$FoM_{unit.} = \frac{{N_{{\rm{Input}}}}}{m}$$

where *N*_Input_ is the number of input combinations the device can process, and m is the number of utilized device units. Compared to the reported state-of-the-art resonator devices^16,21,23^, our proposed resonator design shows almost 10-fold higher computational capability, which is 42.7 data processing per resonator without SNR degradation.

Another critical factor is area efficiency. To uniformly evaluate the area efficiency of these techniques with different feature sizes, the normalized area is calculated by normalizing the actual area with the feature size of the used design technology. Hence, the area efficiency can be calculated by using the following formula:

9 $$f_A = \frac{1}{{{\rm{Area}}\,{\rm{of}}\,{\rm{device/feature}}\,{\rm{size}}^2}}$$

The area efficiency of the proposed device is approximately 1.25 × 10^−3^, which is superior to other reported designs due to its interconnect-free design concept.

A comparison between reported compressors based on other technologies and our proposed resonator compressor is summarized in Table 3. Considering the power consumption of the peripheral circuit (see supporting information), the total power consumption of our tested micro resonator-based compressor is relatively comparable to those reported in the literature^32–37^. Although the area occupation of our proposed device is high, the area efficiency is acceptable compared to other reported designs and can be further improved by properly decreasing the in-plane aspect ratio. In addition, even a single device using our designed resonator shows higher computing power capacity than competing designs; furthermore, each resonator can process 42.7 input vectors due to the improvement of hardware efficiency brought by our paradigm shift into resonator-based computing.

**Table 3:** Comparison between the proposed compressor design and CMOS compressors

Tech.	CMOS 0.35 µm^32^	CMOS 0.18 µm^33^		CMOS 90 nm^34^	CMOS 32 nm^35^	CNTFET 32 nm^36^	Spin-CMOS 20 nm^37^	Des.1 4 µm		Des.4 120 nm
Funct.	4-2 bit	3-2 bit	7-3 bit	4-2 bit	5-2 bit	4-2 bit	4-2 bit	7-3 bit		7-3 bit
Delay	330 ps	70.7 ps	212 ps	178.5 ps	335 ps	116 ps	4 ns	16 ms		0.24 µs
Energy/op	85.8 fJ	43.83aJ	0.27 fJ	2.77 fJ	966 fJ	55 fJ	294 fJ	20.73 pJ	480 nJ^a^	1.14 fJ
Power	260 µW	0.32 µW	1.28 µW	15.5 µW	2885 nW	474 nW	73.4 µW	1.3 nW	30 µW^a^	4.75 nW
Device Count	68 T	20 T	80 T	36 T	58 T	58 T	33 T + 6 M + 3D	3 Res.		3 Res.
$FoM_{unit.}$	0.24	0.4	1.6	0.44	0.55	0.28	0.38	42.67		42.67
Area	1184 µm^2^	–	–	–	5.6 μm^2^	–	–	12,800 μm^2^		0.72 μm^2^
Area Efficiency	10^-4^	–	–	–	1.8 × 10^−4^	–	–	1.25 × 10^−3^		0.02
Process	Post-layout	Prelayout	Prelayout	Prelayout	Post-layout	Prelayout	Fabricated	Fabricated		Simulation

*CNTFET* carbon nanotube field-effect transistor, *T* transistor, *M* magnetic tunnel junction, *D* domain wall strip

^a^Energy consumption considering the peripheral circuit (see supporting information)

Notably, the use of mechanical connections and piezoelectric readout systems is expected to eliminate the need for peripheral circuits in future electromechanical computing systems^38^. Without considering the peripheral circuit, the power of the device fabricated at the 4 µm node is expected to be much lower than those of the compressor devices under all other technical nodes (0.35 µm–20 nm) reported thus far^32–37^. Although many advantages, such as area efficiency and speed, can be achieved by scaling down the devices, other challenges need to be addressed, including device-to-device variations, cost, and difficulty of fabrication. Therefore, an improved nanoscale manufacturing process^39^ and tailored frequency compensating techniques^40,41^ might be necessary to implement nanoscale resonator-based compressors. Moreover, the realization of resonance detection on even smaller scales will present major challenges^31,42^. Therefore, we anticipate that the techniques demonstrated in this work will significantly facilitate the development of multibit computing based on electromechanical resonators.

## Conclusions

This paper demonstrates a design method that combines DC modulation and multiple frequencies to implement a 7-bit resonator based on accumulated arithmetic circuits, presenting the transformation rules for resonance frequency and simplification of binary outputs. Consequently, the implemented interconnect-free compressor offers the prospect of unrivaled integration density, near-zero static power, and low dynamic power because its information does not have to be passed between multiple transistors as occurs in conventional CMOS compressor circuits. The multiple-frequency-driven concept demonstrated here represents the first realization of an interconnect-free 7-bit compressor using only three electromechanical resonators while enabling multibit cascading logic circuits to be executed in a simple and parallel resonator array. It further provides an architecture in which multioperand computing circuits can be easily constructed and reprogrammed. Thus, our resonator-based arithmetic device represents a significant step forward in multioperand programmable mechanical computing for hardware-efficient electronic applications.

## Supplementary information

supplementary
