# Supplementary material for: An interconnect-free micro-electromechanical 7-bit arithmetic device for multi-operand programmable computing
Source: Microsyst Nanoeng. 2023 Apr 3;9:42. doi: 10.1038/s41378-023-00508-0 (PMC10070399; doi:10.1038/s41378-023-00508-0)
Supplement: Supplementary file 1 — supplementary [file 41378_2023_508_MOESM1_ESM.docx]

AN INTERCONNECT-FREE MICRO-ELECTROMECHANICAL 7-BIT ARITHMETIC DEVICE FOR MULTI-OPERANDS PROGRAMMABLE COMPUTING

# Xuecui Zou, Usman Yaqoob, Sally Ahmed, Yue Wang, Khaled Salama, and Hossein Fariborzi

The power consumption with peripheral circuit power consumption is estimated as following steps:

1. Amplifier power consumption

We designed a simple impedance-matching amplifier circuit based on the commercial OPA333 amplifier chip, which can amplify the signal of the developed resonator on-chip. The frequency response by using the developed on-chip amplifier is shown in figure S2, which shows that this amplifier circuit can work for the signal amplification of the resonator. The designed circuit scheme is shown in the figure S1:


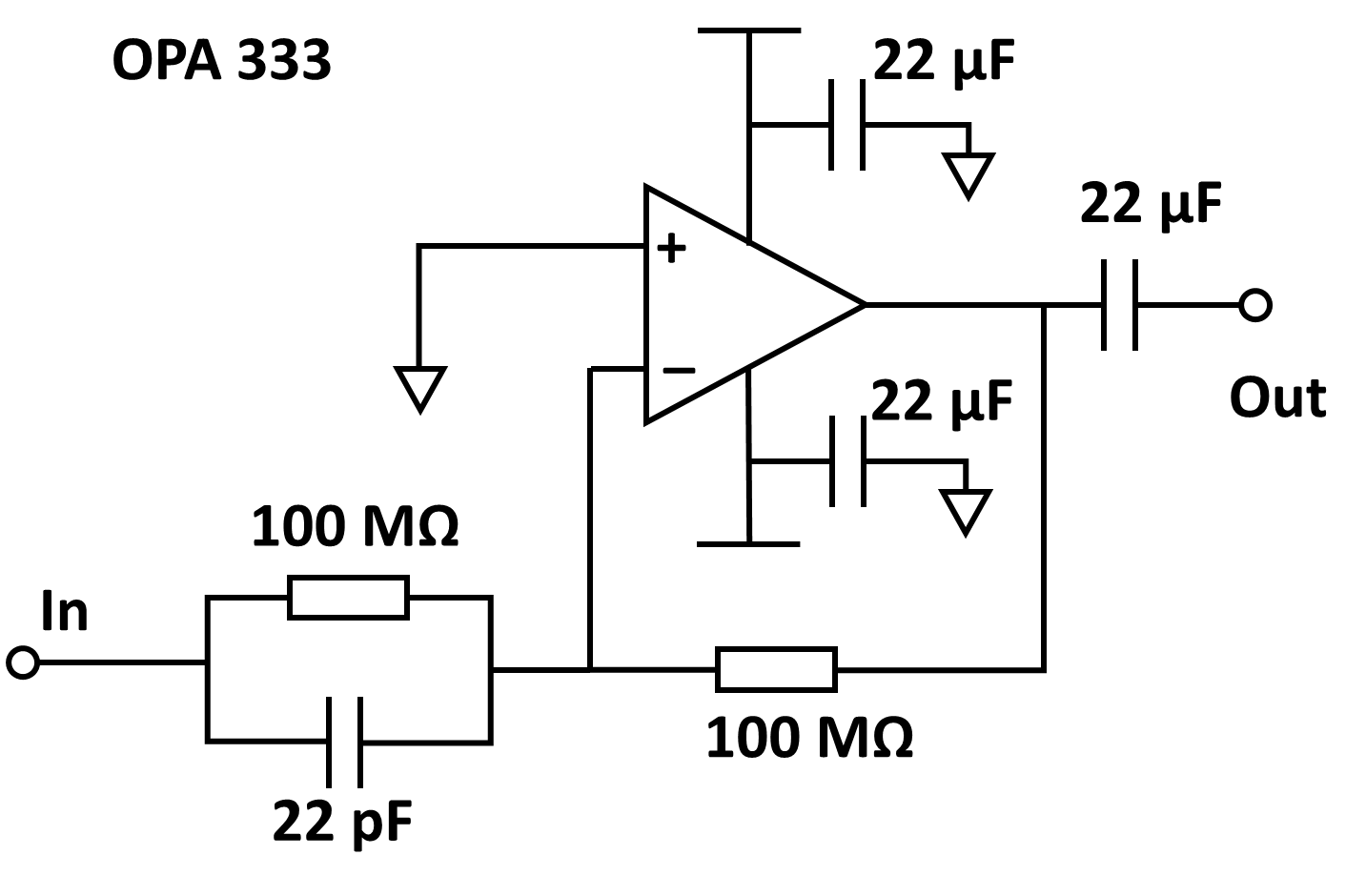

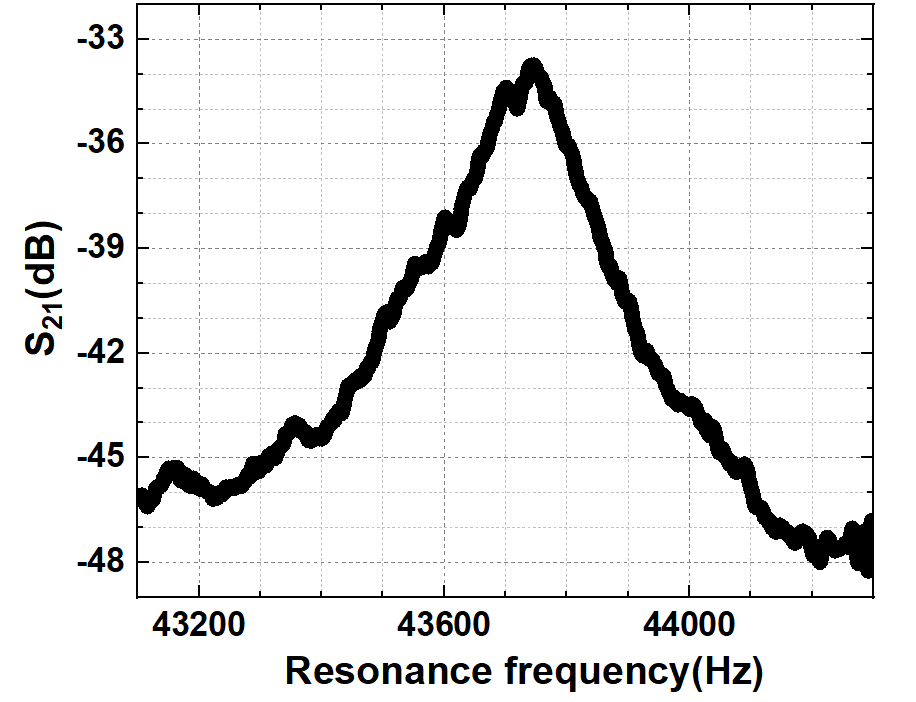


(S1) (S2)

Then the energy consumption of the signal amplification is measured:

$$P_{amp}=V_{DD}I=1.96\left( V \right)*0.01345\left( mA \right)=26.36 \mu W$$

Therefore, the power consumption of 26.36 µW is a relatively accurate estimate of the amplifier energy consumption, which should be added to the total power.

2. Excitation and Measurement consumption

The power consumption of the components from the output port to the input port of the network can be directly measured by the network analyzer based on the equations used in microwave applications characterized by the network analyzer [1]:

$$E_{exc}=E_{out}\left( 1-S_{11}^{2}-S_{21}^{2} \right)={10}^{-6}\left( 1-{0.99953}^{2}-{0.00276}^{2} \right)=9.32\times{10}^{-10}W$$

$$E_{exc}=0.932 nW$$

where $E_{out}$ is the output energy from the source, and$S_{11}$is the reflection coefficient at the input port of the network analyzer, $S_{21}$is forward voltage gain, leading to a power consumption of 0.932 nW.

After taking the peripheral circuit power consumption, the estimated power is:

$$E=E_{exc}+E_{amp}+E_{switch}=30 uW$$

We have updated the comparison table in the revised manuscript, and we put the calculation details of the total power estimation with the peripheral circuit into the supporting information.

References

[1] D. M. Pozar, *Microwave engineering*. John wiley & sons, 2011.
